# Les facteurs pronostiques de survie sans récidive chez les patientes atteintes de tumeur du col de l'utérus

**DOI:** 10.11604/pamj.2015.21.305.5427

**Published:** 2015-08-26

**Authors:** Serpos Dossou, Laurianne James, Hanae Bakkali, Mohammed Afif, Leila Rahali, Joelle Irigo, Etienne Ogandaga, Tayeb Kebdani, Samir Ahid, Noureddine Benjaafar

**Affiliations:** 1 Service de Radiothérapie, Institut National d'Oncologie, CHU Ibn-sina, UM5, Rabat Maroc; 2 Laboratoire de Biostatistique et de Recherche Clinique en Epidémiologie, UM5, Rabat, Maroc; 3 Equipe de Recherche en Pharmaco-épidémiologie, Pharmaco-économie, FMPR, UM5, Rabat, Maroc

**Keywords:** Facteurs pronostiques, survie sans récidive, tumeur cervical, prognostic factors, disease free survival, cervical tumor

## Abstract

La radiochimiothérapie est le traitement de référence des tumeurs du col localement avancées, et plusieurs études ont montré l'importance des facteurs pronostiques sur le contrôle local de la tumeur et la survie des malades. L'objectif de cette étude est d'évaluer l'impact des facteurs pronostiques, en particulier de l'étalement sur la survie sans récidive des patientes suivies pour cancer du col utérin. Il s'agit d'une série rétrospective portant sur 177 femmes suivies en 2011 pour tumeur du col utérin de stade IB à III selon la classification de FIGO 2009 ayant bénéficié d'une radiothérapie à la dose de 46 Grays sur le pelvis et une surimpression sur les paramètres envahis associée à du cisplatine 40mg/m^2^ par semaine suivie de curiethérapie réalisée selon le mode haut débit de dose (HDR) ou bas débit de dose (LDR). La moyenne d'âge était de 53ans, la médiane de l'étalement total était de 65 jours, 75% des patientes ont reçu 4 cures de chimiothérapie, et les patientes ont été suivies après le traitement pendant une durée médiane de 34 mois. La récidive locale et métastatique était de 33,3% chez les patientes ayant des adénopathies pelviennes, contre 16,3% chez celles qui en étaient indemnes (p= 0,031), elle était de 26,3% chez les patientes ayant un étalement supérieur à 65 jours contre 11% chez celles dont l'étalement en était inférieur (p= 0,01). La présence d'adénopathies pelviennes et l'étalement total de la radiothérapie apparaissaient respectivement comme les seuls facteurs pronostiques indépendant de survenue de récidive, p= 0,04 OR= 2,6 IC95% (1,05 6,3) et p= 0,01 OR= 2,9 IC95% (1,26 6,7). En analyse multivariée, la technique de curiethérapie p = 0,003 OR= 0,25 IC95% (0,1 0,6) et l'étalement total du traitement p= 0,0001 OR= 4,7 IC95% (2 10,8) apparaissaient comme les seuls facteurs pronostiques indépendant de survie sans récidive. L'étalement supérieur à 65 jours et la technique de curiethérapie LDR semblent être les facteurs de mauvais pronostic de survie sans récidive dans notre étude.

## Introduction

Le cancer du col de l'utérus est le deuxième cancer de la femme par ordre de fréquence à l'échelon mondial, son incidence est de 13 pour 100000 habitants selon le registre du cancer de Rabat 2006-2008. Depuis l'alerte donnée par la National Cancer Institute en 1999, la radiochimiothérapie concomitante associée à la curiethérapie est le standard dans le traitement des tumeurs invasives du col à un stade localement avancé [1], la méta-analyse de Green avait confirmé le bénéfice de cette radiochimiothérapie sur le contrôle local, la survie sans récidive et la survie globale [2]. La radiothérapie exclusive est réservée pour les formes précoces sans facteurs de mauvais pronostic. La curiethérapie dans les cancers du col utérin est une étape thérapeutique fondamentale, son utilisation est associée à une augmentation du control local de la tumeur. Selon la classification de FIGO 2009, les cancers du col du l'utérus sont classés en divers groupes pronostiques dont la survie à cinq ans diffèrent selon les études [3, 4] cependant outre le stade, d'autres facteurs influenceraient la survie et devraient être pris en compte lors des décisions thérapeutiques [2]. Plusieurs études ont démontré que le cancer du col de l'utérus avait un temps de doublement (Tpot) allant de trois à cinq jours, l'étalement total du traitement fut ainsi identifié comme un facteur pronostic dans les carcinomes du col utérin, cette hypothèse fut confirmée par les études de Fyles qui avait démontré l'importance de la réduction de la durée totale de traitement sur le contrôle local de la tumeur et la survie des patientes traitées par radiothérapie [5, 6]. Le service de radiothérapie de l'institut national d'oncologie de Rabat, dans sa démarche qualité à entrepris à partir de l'année 2011 de réduire le délai d'attente et la durée totale de la radiothérapie des patients devant bénéficier de ce traitement. L'objectif de ce travail est d'évaluer l'impact des facteurs pronostiques, en particulier celui de l'étalement de la radiothérapie sur la survie sans récidive des patientes suivies pour tumeur du col utérin.

## Méthodes

Il s'agit d'une série rétrospective portant sur 177 femmes suivies entre le 1^er^janvier et le 31 décembre 2011 pour tumeur du col utérin de stade IB à III selon la classification de FIGO 2009 histologiquement confirmée, ayant bénéficié d'une radiochimiothérapie concomitante et d'une curiethérapie. Ont été exclues les patientes d'emblées métastatiques, celles qui n'ont pas bénéficié de curiethérapie, et celles qui n'ont pas été suivies dans le service ou perdues de vue juste après le traitement. La radiothérapie a été délivrée à une dose de 46 Grays au point ICRU sur le pelvis, en 23 fractions de 2 Grays par jour, cinq jours par semaine, aux photons d'un accélérateur linéaire de haute énergie (18 MV). Les techniques conventionnelles bidimensionnelles par deux faisceaux antérieur et postérieur ou conformationnelles tridimensionnelles par 4 faisceaux en boite ont été utilisées avec 2/3 de la dose apportée par les faisceaux antérieur et postérieur, et le 1/3 par les faisceaux latéraux. Un complément d'irradiation de 9Gray (3x3Gy) ou 10 Gray (5x2Gy) sur les paramètres envahis à été effectué soit immédiatement après la première série, soit après la curiethérapie. Les adénopathies macroscopiques (>1cm) pelviennes et lomboaortiques ont reçus également un complément d'irradiation de 15 à 20 Gray. La chimiothérapie a été réalisée en concomitant avec la radiothérapie avec du cisplatine à la dose de 40 mg/m^2^/semaine sous contrôle d'une bonne tolérance rénale et hématologique, elle a été adaptée en fonction de la clearance de la créatinine et reportée en cas d'anémie ou de mauvaise tolérance.

La curiethérapie utéro-vaginale a été effectuée après la radiothérapie chez toutes les patientes à la dose unique de 24 Grays pour le bas débit de dose (LDR) et selon plusieurs fractionnements pour le haut débit de dose (HDR). Les prescriptions ont été faites au point A pour la curiethérapie haut débit de dose avec les fractionnements suivant: 3x8 Grays, ou 4x7Grays, en s'assurant que la dose au point A est supérieure à 90 Grays. Les applicateurs type Fletcher et d'autres adaptés à l'anatomie de chaque patiente ont été utilisés, le radio-élement était de l'iridium 192. L'étalement total du traitement avait inclus la durée de la radiothérapie, l'intervalle entre la radiothérapie et la curiethérapie, la durée de cette curiethérapie et la durée du complément d'irradiation sur les paramètres et les adénopathies en cas de besoin. Les patientes ont été suivies une fois par semaine au cours de la radiochimiothérapie et tous les trois mois pendant deux ans et tous les six mois à partir de la troisième année après la fin du traitement. La récidive a été suspectée par l'examen clinique, confirmée par le scanner ou l'IRM et par une nouvelle biopsie. La survie sans récidive était définie par le temps écoulé entre le début du traitement et l'événement à l'origine de la récidive locale ou métastatique (date de la biopsie de confirmation, date de récidive à l'IRM, ou au scanner, date du décès pour les patientes décédées avant toute récidive authentifiée). Les variables catégorielles ont été comparées en utilisant le test de Khi2, les facteurs influençant l'étalement et les facteurs pronostiques de récidive ont été analysés par une régression logistique binaire. La survie sans récidive a été analysée par la méthode de Kaplan-Meier, les comparaisons ont été faites avec le test du log-rank et le modèle de cox, avec un intervalle de confiance de 95 %. Les résultats ont été considérés comme significatifs au seuil **α** fixé à p < 0,05 et les analyses statistiques ont été réalisées en utilisant la version 20 du logiciel SPSS. La date de pointe a été fixée au **15 Avril 2014**.

## Résultats

Cent soixante dix sept (177) patientes étaient éligibles, la moyenne d'âge était de 53,1 ± 11,5 ans, la durée médiane du déroulement de la radiothérapie était de 38 (36-42) jours avec une minima de 30 jours, le délai médian entre la radiothérapie et la curiethérapie était 18 (13-33) jours avec une minima de 1 jour et la médiane de l'étalement total était de 65 (56-83) jours avec une minima de 40jours, 75% des patientes ont reçu 4 cures de chimiothérapie, et les patientes ont été suivies après le traitement pendant une durée médiane de 34 (30-36,5) mois, les caractéristiques de la population sont résumées dans le Tableau 1. L'étalement était plus long (> 65 jours) chez les patientes ayant bénéficié d'une radiothérapie bidimensionnelle par rapport à celles qui ont bénéficié d'une radiothérapie tridimensionnelle (71,7 vs 44,4%), avec p < 0,001, de même les patientes ayant bénéficié d'une curiethérapie haut débit de dose avaient plus un étalement supérieur à 65 jours par rapport à celles qui ont reçu une curiethérapie bas débit de dose (91,5 vs 34,7%) avec p <0,001. En analyse multivariée, la technique de curiethérapie haut débit de dose multipliait par 21 la probabilité d'avoir un étalement supérieur à 65 jours, Tableau 2.

**Tableau 1:** Caractéristiques de la population

Caractéristiques	Valeur N= 177
- Stades	
IB	23 (13)
IIA	8 (4,5)
IIB	92 (52)
III	54 (30,5)
- Taille tumorale	
< 4cm	70 (39,5)
> 4cm	107(60,5)
- Atteinte paramétriale	146 (82,5)
- Adénopathies pelviennes	30 (16,9)
-Adénopathies lomboaortiques	5 (2,8)
- Hydronéphrose	9 (5,1)
- Anémie	18(10,2)
- Histologie	
Carcinome epidermoide	169(95,5)
Adénocarcinome	7 (4)
Autre	1 (0,6)
- Type de radiothérapie	
3D	117(66,1)
2D	60(33,9)
- Type de curiethérapie	
LDR	118(66,7)
HDR	59(33,3)
- Statut	
Pas de récidive	143(80,8)
Récidive	34(19,2)

Les valeurs sont exprimées eu effectif (pourcentage)

**Tableau 2:** Facteurs influençant l’étalement

	Analyse univariée			Analyse multivariée		
	OR	95%IC	p	OR	95%IC	p
Age	0,9	(0,5 1,6)	0,7	1,03	(0,5 2)	0,9
Stades	1,1	(0,8 1,4)	0,7	1,4	(0,9 2)	0,2
Adp pelviennes	1,2	(0,5 2,5)	0,7	1,7	(0,6 4,8)	0,3
Adp LAo	0,6	(0,1 3,5)	0,5	0,8	(0,1 6,5)	0,9
Hydronéphrose	0,4	(0,1 1,7)	0,2	0,3	(0,05 1,8)	0,2
Anémie	1,1	(0,4 3)	0,9	0,9	(0,25 3,2)	0,9
Type de Rth	3,1	(1,6 6,1)	**0,001**	1,6	(0,7 3,6)	0,3
Type de Crth	20,3	(7,5 54)	**0,001**	21,1	(7,4 61)	**0,0001**

**Rth** = Radiothérapie, Crth = Curiethérapie, **Adp** = Adénopathie**, LAo** = Lomboartique, **OR** = Odds ratio **IC95%** = Intervalle de confiance 95%

**Figure 1:**
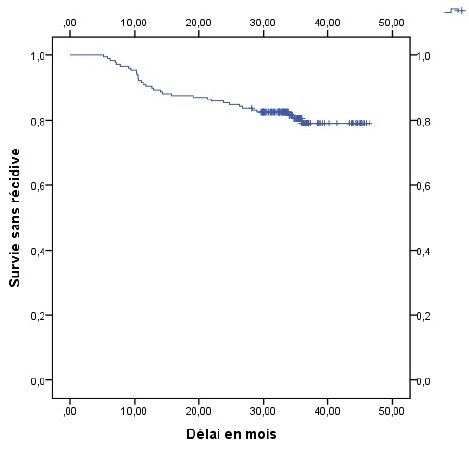
Survie sans récidive

**Figure 2:**
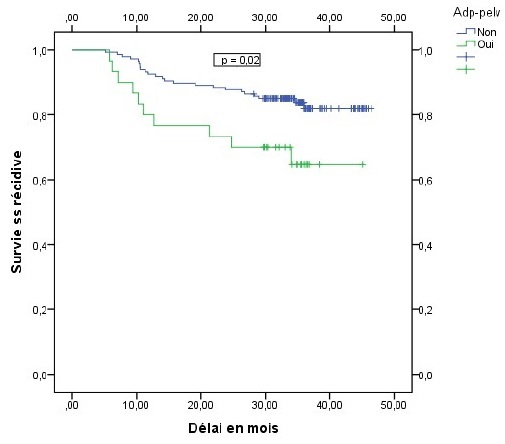
Survie sans récidive en fonction des adenopathies

**Figure 3:**
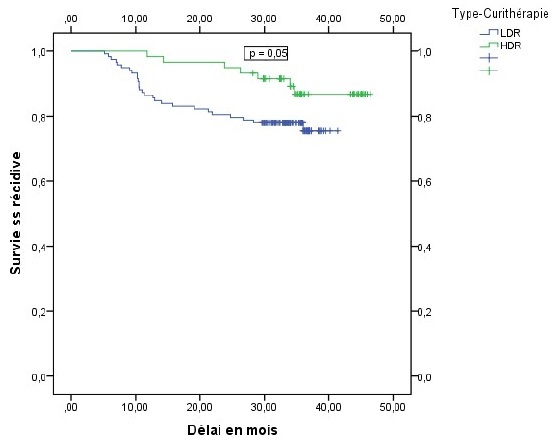
Survie sans récidive en fonction du type de curiethérapie

**Figure 4:**
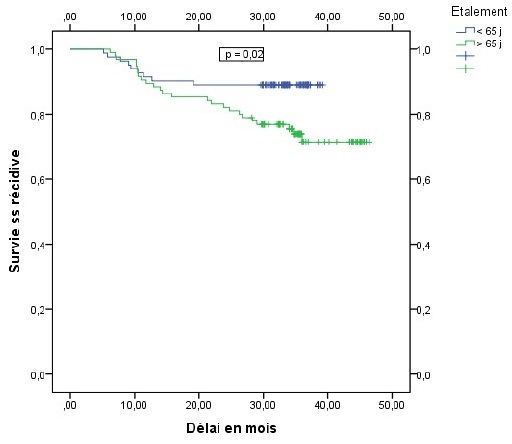
Survie sans récidive en fonction de l’étalement

La récidive locale et métastatique était de 33,3% chez les patientes ayant des adénopathies pelviennes contre 16,3% chez celles qui en étaient indemnes (p = 0,031), elle était de 26,3% chez les patientes ayant un étalement supérieur à 65 jours contre 11% chez celles dont l'étalement en était inférieur (p = 0,01). La présence d'adénopathies pelviennes et l'étalement total de la radiothérapie apparaissaient comme les seuls facteurs pronostiques indépendant de survenue de récidive. Le risque de récidive était alors multiplié par 2,6 en présence d'adénopathie pelvienne de façon statistiquement significative p = 0,04 OR = 2,6 IC95% (1,05 6,3), ce risque était aussi multiplié par 2,9 pour toute augmentation d'une journée de l'étalement au delà de 65 jours, p = 0,01 OR = 2,9 IC95% (1,26 6,7). Le délai moyen de survie sans récidive était estimé à 40 mois et la survie sans récidive à 3 ans était de 80 % tout stade confondu (Figure 1). En analyse univariée, la survie sans récidive à 3 ans était de 82% chez les patientes indemnes d'adénopathies pelviennes contre 64,6% chez les patientes qui avaient des adénopathies envahies (p = 0,02), elle était de 86,7% chez les patientes ayant bénéficié d'une curiethérapie haut débit de dose (HDR) contre 75,5% chez les patientes traitées par curiethérapie bas débit de dose (LDR) p = 0,05 (Figure 2, Figure 3). Cette survie sans récidive à 3 ans était de 89% chez les patientes dont l'étalement était inférieur à 65 jours contre 71,4% chez les patientes dont l'étalement en était supérieur p = 0,02 (Figure 4). En analyse multivariée, la technique de curiethérapie (p = 0,003) et l'étalement total du traitement (p = 0,0001) apparaissaient comme les seuls facteurs pronostiques indépendants de survie sans récidive. Il n'existait pas de différences statistiquement significatives de survie en tenant compte de l'âge, de l'anémie, de l'hydronéphrose, des adénopathies pelviennes et lomboaortiques de la taille et du stade tumoral, de la technique de radiothérapie et de l'histologie de la tumeur, Tableau 3. Les toxicités aigues répertoriées étaient digestives à type de diarrhées (2,5%) nausées et vomissements (68%), puis les toxicités urinaires à type cystites aigues (43%) et enfin les toxicités cutanées à type de radiodermite (11%).

**Tableau 3:** Facteurs pronostiques de survie sans récidive

	Log-rank		Cox's	
	p	HR	95%IC	p
Age	0,8	1,3	(0,6 2,7)	0,4
Stade	0,3	0,9	(0,6 1,6)	0,9
Paramètre	0,6	0,6	(0,1 3,5)	0,5
Taille	0,4	1,3	(0,5 2,9)	0,6
Anémie	0,7	1,6	(0,5 5)	0,4
Hydronéphrose	0,4	0,4	(0,5 6)	1,7
Histologie	0,8	0,3	(0,4 2,5)	0,3
Adénopathie pelviennes	**0,02**	2,1	(0,9 4,9)	0,08
Adénopathie lomboaortiques	0,23	1,06	(0,2 5,3)	0,9
Type de radiothérapie	0,6	0,9	(0,4 2,1)	0,9
Type de curiethérapie	**0,05**	0,25	(0,1 0,6)	**0,003**
Etalement	**0,02**	4,7	(2 10,8)	**0,0001**

**Log-rank** = Test de log-rank **Cox's =** Modèle de cox

## Discussion

Le traitement de choix des tumeurs localement avancées du col utérin est la radiochimiothérapie suivie de curiethérapie, et plusieurs facteurs pronostiques influençant la survie ont été mis en évidence, certains sont liés aux caractéristiques des patients (âge, taux d'anémie), d'autres liés à la tumeur (stade, taille, atteinte ganglionnaire, l'histologie) et enfin d'autres liés aux caractéristiques du traitement (étalement, la technique d'irradiation) [7]. Des études ont suggéré que l'âge était un facteur pronostic défavorable de la survie [8], car l'analyse de survie avait révélé que les patientes plus jeunes avaient une altération de survie dans la cohorte, cependant les données d'autres auteurs surtout ceux qui ont analysé le traitement chirurgical des tumeurs cervicales ont montrés que l'âge n'était pas le facteur pronostic aggravant la survie [9]. Cette controverse s'expliquait par le fait que les femmes jeunes avec une tumeur de petite taille bénéficiaient d'une chirurgie, alors les femmes de même âge ayant de grosses tumeurs étaient traitées par la radiothérapie, par conséquent le mauvais pronostic dans le groupe des patientes jeunes pourrait être lié à des facteurs autres que l'âge, la taille tumorale par exemple. Dans notre cohorte l'âge n'apparaissait pas comme un facteur pronostic de survie sans récidive.

L'anémie en cours de traitement par radiothérapie était décrite comme l'une des causes de l'échec des traitements, et selon Grigiene R l'anémie était un facteur pronostic indépendant de survie sans maladie [10]. Dans notre cohorte, l'anémie ne semble pas influencer la survie sans récidive, probablement parce que les patientes étaient transfusées avant le début de la radiothérapie. Le mécanisme de la relation entre l'anémie et le mauvais pronostic des patientes atteintes du cancer du col utérin n'est pas clair, l'une des hypothèses était que les patientes ayant un mauvais pronostic, étaient anémiées au moment du diagnostic, et que l'anémie liée à la tumeur était plutôt un signe d'agressivité tumorale comme la perte de poids et l'état général [11] dans ces cas, la correction du taux d'hémoglobine durant le traitement n′aura aucun impact. L'autre hypothèse est que l'hypoxie tissulaire est un facteur de radiorésistance, et l'anémie aggrave l'hypoxie, dans ce cas la correction de l'anémie devrait améliorer les résultats du traitement [12]. Le stade FIGO est le facteur pronostic le plus important des patientes diagnostiquées pour cancer du col utérin, et de nombreux autres facteurs pronostiques connus sont calqués sur cette classification essentiellement basée sur l'examen physique, et qui est à l'origine parfois de sous stadification [13]. Ce facteur pronostic ne semble pas influencer la survie sans récidive dans notre série. La taille tumorale est un paramètre pronostic bien établi indépendant du stade, et s'était reflété dans la classification initiale de FIGO pour les stades IA-IB2. Dans la classification de FIGO 2009, la taille tumorale a été ajouté en tant que paramètre pour distinguer les tumeurs IIA1 (< 4cm) et IIA2 (> 4 cm) en raison de sa valeur pronostic considérable [13, 14]. Cependant ce facteur pronostic ne semble pas influencer la survie sans récidive dans notre série probablement à cause des variabilités d'appréciation à l'examen clinique. L'atteinte paramétriale influencerait la survie sans récidive dans les stades localisés [14] ce facteur ne semble pas influer la survie sans récidive dans notre série. L'atteinte ganglionnaire à un impact péjoratif sur la survie sans récidive dans notre série, ce facteur pronostic influence également la survie sans récidive dans la série de Grigiene [10]. La valeur pronostique de l'histologie des tumeurs cervicales sur la survie a été activement débattu, une étude récente à montré que les adénocarcinomes étaient plus agressifs et étaient associées à une diminution de la survie des stades précoces et avancés [15]. L'histologie ne semble pas influencer la survie sans récidive dans notre série probablement à cause du faible nombre des adénocarcinomes.

La méta analyse de Gustavo avait montré qu'il n'y avait pas de différence entre les techniques de curiethérapie haut débit de dose (HDR) et bas débit de dose (LDR) en terme de survie globale, et de récidive locale [16]. Cependant en analyse multivariée la curiethérapie LDR apparaissait dans notre cohorte comme un facteur indépendant de mauvais pronostic en terme de survie sans récidive. Cette différence s'expliquerait probablement par le fait des erreurs dues à l'insertion inapproprié des applicateurs, ou une imagerie inadéquate pouvant identifier le mauvais placement, entrainant ainsi une irradiation inappropriée et par conséquent un risque élevé de récidive. Une autre explication serait le manque de stabilité et de précision de l'applicateur due aux mouvements des patientes qui restaient appliqués pendant deux à trois jours lors de la curiethérapie LDR augmentant ainsi le risque d'incertitude, c'est l'une des raisons pour lesquelles la curiethérapie haut débit de dose (HDR) est préférée actuellement. Plusieurs études (Perez, Girinsky) ont montrés que l'étalement total était un facteur pronostic de survie sans récidive et de survie globale pour les patientes traitées par radiothérapie seule, Withers avait montré que la repopulation clonogène était accélérée 28 jours après le début de la radiothérapie en fractionnement classique chez les humains, il avait conclu qu'au delà de 4 semaines d'irradiation qu'une dose supplémentaire de 0,61 gray par jour était nécessaire pour surmonter la prolifération de la veille [17]. Selon Fyles [6] la perte du control local serait approximativement de 1% par jour quand le traitement est prolongé au delà de 30 jours, mais cette repopulation serait compensée par la delivrance de forte dose lors de la curiethérapie dans le cas du traitement du cancer du col. Pour Girinsky [18] cette perte du contrôle local serait de 1,1% par jour à partir d'une durée de traitement excédant 52 jours et 0,9% par jour pour un étalement excédant 55 jours dans la série de Perez [19]. Pour Lanciano [20] le taux de récidive à 4 ans passait de 6 à 20% lorsque l'étalement variait d'une durée inférieure ou égale à 6 semaines à une durée supérieure ou égale à 10 semaines (p = 0,0001), la survie était de 65% et 54% en 5ans quand l'étalement était inférieur à 55 jours versus supérieur à 55 jours dans la série de Petereit [21], dans notre série la survie sans récidive à 3 ans passait de 89 à 71,4% quand l'étalement était respectivement inférieur à 65 jours et supérieur à 65 jours, cet étalement de 65 jours était comparable à celui de Chen qui était de 63 jours [22]. Pour Girinsky [18] le risque d'échec local était multiplié par 2,4 lorsque l'étalement passait de 52 jours à 62 jours, dans notre série le risque de récidive était multiplié par 2,9 pour toute augmentation d'une journée de l'étalement au delà de 65 jours. Les limites de notre étude reposent sur son caractère rétrospectif, ne permettant pas ainsi de recueillir les toxicités de toutes les patientes, à l'impossibilité d'apprécier la réponse tumorale après la radiothérapie et l'impossibilité d'apprécier avec précision le débit de dose lors de la curiethérapie.

## Conclusion

La survie sans récidive locale ou métastatique lors du traitement du cancer du col localement avancé par radiochimiothérapie concomitante est tributaire d'un certain nombre de facteurs pronostiques, dans notre série l'étalement total du traitement et la technique de curiethérapie LDR sont les facteurs pronostiques de survie sans récidive retrouvés. Une étude prospective est souhaitable pour confirmer ces hypothèses.
